# Degree of conversion and bond strength of resin-cements to feldspathic ceramic using different curing modes

**DOI:** 10.1590/1678-77572016-0221

**Published:** 2017

**Authors:** Veridiana Resende NOVAIS, Luís Henrique Araújo RAPOSO, Rafael Resende de MIRANDA, Camila de Carvalho Almança LOPES, Paulo Cézar SIMAMOTO, Carlos José SOARES

**Affiliations:** 1 Universidade Federal de Uberlândia, Faculdade de Odontologia, Departamento de Dentística e Materiais Odontológicos, Uberlândia, MG, Brasil.; 2 Universidade Federal de Uberlândia, Faculdade de Odontologia, Departamento de Oclusão, Prótese Fixa e Materiais Odontológicos, Uberlândia, MG, Brasil.; 3 Universidade Federal de Uberlândia, Faculdade de Odontologia, Uberlândia, MG, Brasil.

**Keywords:** Polymerization, Resin cements, Shear strength

## Abstract

Resin cements have led to great advances in dental ceramic restoration techniques because of their ability to bond to both dental structures and restorative materials.

**Objective:**

The aim of this study was to assess the performance of resin cements when different curing modes are used, by evaluating the degree of conversion and bond strength to a ceramic substrate.

**Material and Methods:**

Three resin cements were evaluated, two dual-cured (Variolink II and RelyX ARC) and one light-cured (Variolink Veneer). The dual-cured resin cements were tested by using the dual activation mode (base and catalyst) and light-activation mode (base paste only). For degree of conversion (DC) (n=5), a 1.0 mm thick feldspathic ceramic disc was placed over the resin cement specimens and the set was light activated with a QTH unit. After 24 h storage, the DC was measured with Fourier transform infrared spectroscopy (FTIR). For microshear bond strength testing, five feldspathic ceramic discs were submitted to surface treatment, and three cylindrical resin cement specimens were bonded to each ceramic surface according to the experimental groups. After 24 h, microshear bond testing was performed at 0.5 mm/min crosshead speed until the failure. Data were submitted to one-way ANOVA followed by Tukey test (p<0.05). Scanning electron microscopy (SEM) was used for classifying the failure modes.

**Results:**

Higher DC and bond strength values were shown by the resin cements cured by using the dual activation mode. The Variolink II group presented higher DC and bond strength values when using light-activation only when compared with the Variolink Veneer group.

**Conclusion:**

The base paste of dual-cured resin cements in light-activation mode can be used for bonding translucent ceramic restorations of up to or less than 1.0 mm thick.

## Introduction

The ability of ceramics to match natural dentition, due to their good physical and optical properties, makes them the material of choice for patients with high esthetic expectations^17^. Conservative ceramic restorations for changing the position, shape, or color of anterior teeth have been widely used in modern dental practice^1^. The success of ceramic veneers has been attributed to the establishment of a durable bond among tooth tissues, luting composite and ceramic substrate^18^.

Resin-based cements are the materials most commonly used for luting dental ceramic restorations, mainly because of their improved physicochemical properties. In general, these materials are composed of a polymeric matrix based on dimethacrylate monomers, filler particles and pigments^9^. Commercially available resin cements for luting ceramic veneers can be light activated or dual-cured, depending on the opacity and thickness of the ceramic restoration^28^. Light-cured resin cements allow extended working time and removal of the excess around the restoration before light-activation, reducing the time needed for finishing after restorations have been luted^20^. In addition to these features, light-cured resin cements have the great advantage of improved color stability, since no tertiary amines are used as chemical activator, which could cause color change over the time^28^.

Dual-cured resin cements were introduced to combine the advantages of light- and chemically-cured resin cements and are the most common luting materials used nowadays^1^. These systems are composed of a catalyst paste consisting of benzoyl peroxide (initiator) and base paste, containing a light-cured resin cement, in an endeavor to increase free radical concentration even under thick or opaque restorations^28^. The free radicals formed by the chemical reaction (tertiary amines/benzyl peroxide) would compensate for the lack of those that result from the physical initiation system activated by light (aliphatic amines/camphorquinone)^11^. However, dual-cured resin cements may have reduced color stability, due to the possibility of some degree of oxidation occurring in the components involved in chemical curing (tertiary amines)^16^.

Therefore, light-cured resin cements may be a more suitable option when luting thin translucent ceramic veneers^29^. Nonetheless, some dual-cured resin cement manufacturers offer a light-cured option for their systems in the absence of a light-cured resin cement, and indicate that dental practitioners should use the base paste only in a light-activation mode. However, there is still no evidence that the light-cured components of dual-cured resin cements have the ability to reach good physical and mechanical properties when used alone.

In addition to formulation, the success of ceramic restorations depends on optimal cure of resin cements. The degree of conversion is crucial in determining the mechanical performance of resin-based materials and their biocompatibility. The strength, elastic modulus, hardness and solubility of composite resins are directly related to the degree of conversion^11^. As seen, inadequate curing with a reduced degree of conversion may cause changes in the physical characteristics of resin-based components, affecting their mechanical properties, altering dimensional stability, decreasing bonding to tooth structures, capable of resulting in the unsatisfactory clinical performance of these materials^25^.

Therefore, the aim of this study was to assess the performance of resin cements when using different curing modes, by evaluating the degree of conversion and bond strength to a ceramic substrate. The hypothesis was that dual-cured resin cements used in the dual activation mode with base and catalyst pastes, would behave differently when using these cements in the light-activation mode with base paste only. A resin cement with a light-curing option only was also used for the comparisons.

## Material and methods

For this study, three resin cements were evaluated, two dual-cured [Variolink II (VL), shade A3, Ivoclar Vivadent; Schaan, Liechtenstein; and RelyX ARC (RX), shade A3, 3M-ESPE; St. Paul, MN, USA], and one light-cured [Variolink Veneer (VL-V), shade High Value +3, Ivoclar Vivadent]. Dual-cured resin cements were tested in the dual activation mode by mixing the base and catalyst pastes (BC), or in the light-activation mode, by using the base paste only (B). The light-cured resin cement was tested only in the light-activation mode. Thus, five experimental groups were defined.

**Figure 1:**
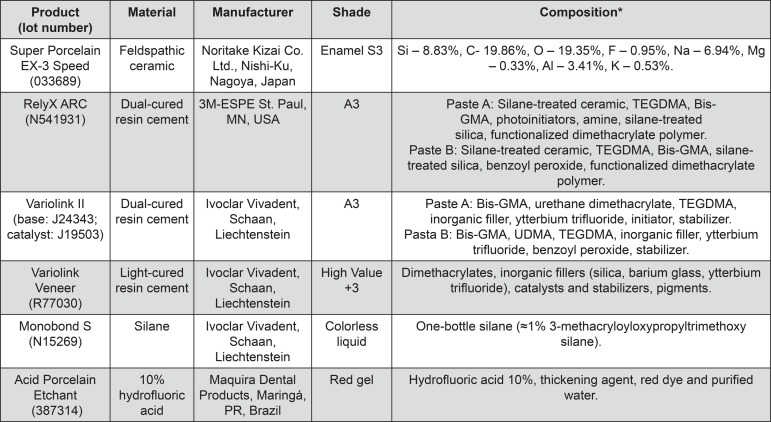
Materials used in this study

Twenty-six feldspathic ceramic discs (Super Porcelain EX-3 Speed, Enamel S3, Noritake Dental Supply Co Ltd; Nagoya, Aichi, Japan) were obtained, one of which was used to perform the degree of conversion test and 25 were used for microshear bond strength testing. The materials information is summarized in Figure 1. The ceramic discs were produced in a stainless steel split matrix (12.5 mm in diameter x 1.2 mm thick). The ceramic powder/liquid ratio was used according to the manufacturer’s instructions and the mixture was placed in the mold. The ceramic discs were submitted to a sintering cycle in an appropriate furnace (Alumini Press II, EDG; São Carlos, SP, Brazil), according to the firing schedule suggested by the manufacturer. In the conclusion of the procedure, ceramic disc dimensions were 10 mm in diameter and 1 mm thick.

*According to manufacturers. Si - silicon; C – carbon; O – oxygen; F – fluorine; Na – sodium; Mg – magnesium; Al – aluminum; K – potassium; Bis-GMA: 2,2-bis[p-(2’-hydroxy-3’methacryloxypropoxy)phenyl]propane; TEGDMA: triethylene glycol dimethacrylate; UDMA: 1,6-bis(methacryloxy-2-ethoxycarbonylamino)-2,4,4-trimethylhexane

### Degree of conversion

**Figure 2:**
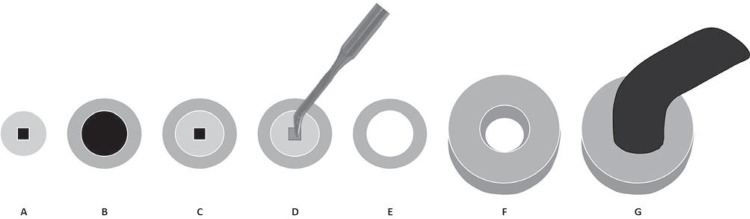
Preparation of specimens for degree of conversion: A- Vinyl mold; B- Teflon mold; C- Teflon mold positioned around vinyl mold; D- Insertion of resin cement into vinyl mold; E- Ceramic disc positioned over the resin cement specimen; F- Teflon ring positioned around the ceramic disc; G- Light-curing of resin cement through ceramic disc using a QTH unit

For testing the degree of conversion (DC), resin cements were put into vinyl molds (1 mm thick x 2 mm inner diameter x 10 mm outer diameter). A Teflon mold was placed around the vinyl molds. Mylar strips (Quimidrol; Joinville, SC, Brazil) were used on bottom and top surfaces of the mold to ensure smoothness of the specimens, allow the ceramic disc to be placed over the resin cement and to avoid inhibition of polymerization by oxygen^4^. The ceramic disc and the tip of the light-curing unit were positioned over the resin cement specimens with the help of a Teflon ring. The diameter of the light wand tip was exactly the same diameter as that of the disc/sample. For the dual activation mode, the base and catalyst pastes of dual-cured resin cements were mixed according to the manufacturer’s directions and then three minutes were waited for the self-cure reaction to occur. After this, all the groups were light-cured through the ceramic discs using a quartz-tungsten halogen (QTH) light-curing unit (Optilux 501, Kerr; Orange, CA, USA) at 800 mW/cm^2^ output for 40 s (Figure 2). All specimens were stored under dry and dark conditions at 37°C for 24 h. The DC of the specimens (n=5) was determined using attenuated total reflectance/Fourier transform infrared spectroscopy (ATR/FTIR, Vertex 70, Bruker; Ettlingen, Baden-Württemberg, Germany). A preliminary readout of each uncured material was recorded in absorbance spectrum acquired by scanning the specimens 32 times over a range from 4000 to 400 cm^-1^ with a resolution of 4 cm^-1^. An additional spectrum was acquired of the cured specimen after dry storage at 37°C for 24 h, shielded from ambient light. The DC was calculated according to the following formula, using the absorption peak of the C=O ester groups as reference, because no aromatic peaks were identified in Variolink Veneer:

% DC = 100 [1 - (Aa_(C=C)_Ab_(C=O)_ / Ab_(C=C)_Aa_(C=O)_)]

### Microshear bond strength

Twenty-five ceramic discs were embedded in PVC cylinders using polyester resin (AM 190 resin, AeroJet; São Paulo, SP, Brazil). After divesting, the ceramic disc surfaces were sequentially polished using silicon-carbide papers (#600, 800, 1200 and 2000 grit sizes; Norton; Campinas, SP, Brazil), mounted on a water-cooled polisher (Aropol 2V, Arotec S/A; São Paulo, SP, Brazil). The discs were submitted to surface treatment with 10% hydrofluoric acid (Maquira Dental Products; Maringá, PR, Brazil) for 2 min^27^. The acid was removed with air/water spray and the specimens were cleaned in an ultrasonic bath (USC1400, Unique; Indaiatuba, SP, Brazil) with distilled water for 5 min. After drying the specimens, a layer of silane coupling agent (Monobond S, Ivoclar Vivadent) was applied and left to react for 1 min.

**Figure 3:**
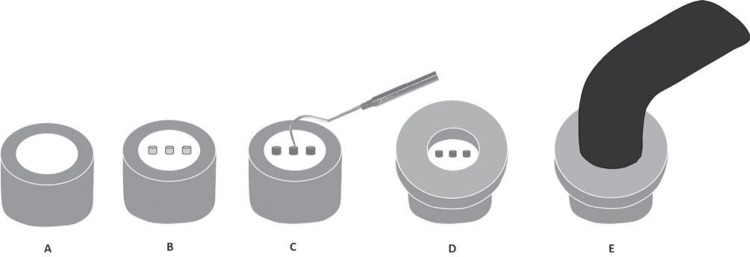
Preparation of specimens for microshear bond strength: A- Ceramic disc embedded in polyester resin cylinder; B- After surface treatment, three Tygon tubes were placed on each ceramic disc; C- Insertion of resin cement into Tygon tubes; D- Teflon ring positioned over the ceramic disc and around Tygon tubes; E- Light-curing of resin cement specimens using a QTH unit

The discs were randomly divided according to the five experimental groups. Three Tygon tubes (0.5 mm high x 0.75 mm in diameter) were placed in parallel on each ceramic disc and filled with resin cement. For the dual activation mode, the cement was light-activated three minutes after it was inserted^24^. The specimens were light-cured by using the QTH unit at 800 mW/cm^2^ output for 40 s, with the light source placed at the top of the specimens, and within a Teflon ring, thus light-curing all the resin cylinders simultaneously (Figure 3). All specimens were stored under relative humidity and dark conditions at 37°C for 24 h, and after storage, the Tygon tubes were removed using a scalpel blade No. 12.

Microshear testing (n=5) was performed in a mechanical testing machine (Microtensile OM100, Odeme Dental Research; Luzerma, SC, Brazil), in which each ceramic disc was attached to a flat base by means of cyanoacrylate-based glue (Super Bonder, Loctite; Itapevi, SP, Brazil). A stainless steel wire (0.2 mm in diameter) (Morelli Ortodontia; Sorocaba, SP, Brazil) was placed around the resin cement cylinder at its base, as close as possible to the ceramic/resin cement interface. The test was performed at a crosshead speed of 0.7 mm/min until failure of the specimens.

### Failure mode analysis

The failure mode of the specimens was analyzed by scanning electron microscopy (SEM) (TM3000, Hitachi High-Technologies Co.; Tokyo, Kantō, Japan) at 200x magnification, at an acceleration voltage of 25 kV. The failure modes were classified as: I- adhesive failure; II- cohesive failure at resin cement; III- cohesive failure at ceramic substrate; IV- mixed failure with predominance of resin cement; V- mixed failure with predominance of ceramic substrate. Adhesive failure was characterized as a fault at the junction between the ceramic and the resin cement; cohesive failure when there was a fracture in the body of the cement or the ceramic; and mixed failure when there was cohesion failure in both materials, simultaneously.

### Statistical analysis

Degree of conversion and microshear bond strength data were individually analyzed by one-way analysis of variance (ANOVA). Tukey *post hoc* tests were performed to determine differences among groups. Statistical significance was set at α=0.05. Descriptive analysis was used for failure mode.

## Results

### Degree of conversion

The mean degree of conversion (%) and standard deviation obtained for the experimental groups are shown in Table 1. Significant differences were observed among the groups (p<0.001). The resin cements used in the dual activation mode (base and catalyst) showed higher DC values. The base paste of the dual-cured cements used in the light-activation mode presented intermediate DC values. The light-cured resin cement showed lower DC values when compared with the other cements.

**Table 1:** Mean degree of conversion (%) and standard deviation for the experimental groups

Resin Cement	Degree of Conversion (%)
RX-BC	72.8 (3.7)^A^
RX-B	57.5 (4.4)^C^
VL-BC	65.7 (2.9)^B^
VL-B	58.3 (2.1)^C^
VL-V	48.6 (4.1)^D^

Means followed by different letters indicate statistically significant difference (p<0.05)

### Microshear bond strength

The mean bond strength values and standard deviation are shown in Table 2. The Variolink II groups, activated in dual mode or light-activation mode only showed the highest bond strength values, with similar results between them; and were similar to those of RelyX ARC in dual activation mode. The light-cured resin cement Variolink Veneer presented similar values to those of RelyX ARC in the dual activation mode and also to RelyX ARC in the light-activation mode.

**Table 2:** Mean bond strength (µSBS) values and standard deviation for the experimental groups

Resin Cement	Bond Strength
RX-BC	33.5 (2.8)^AB^
RX-B	27.8 (4.6)^C^
VL-BC	36.5 (4.7)^A^
VL-B	36.0 (2.0)^A^
VL-V	29.8 (2.0)^BC^

Means followed by different letters indicate statistically significant difference (p<0.05)

### Failure mode

**Figure 4:**
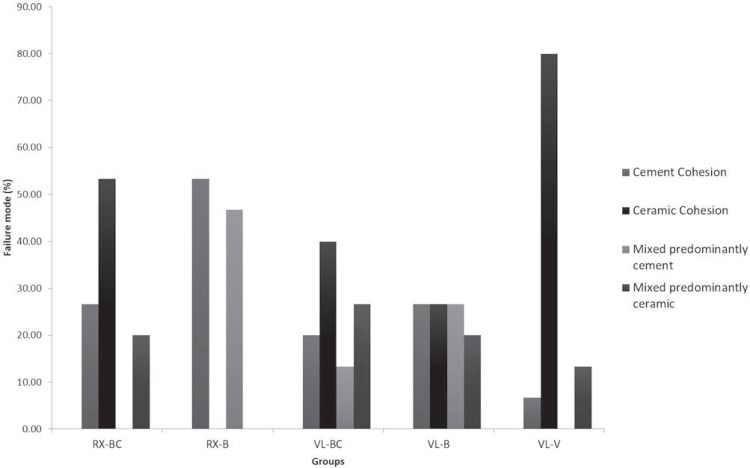
Failure mode distribution (%) for the experimental groups

**Figure 5:**
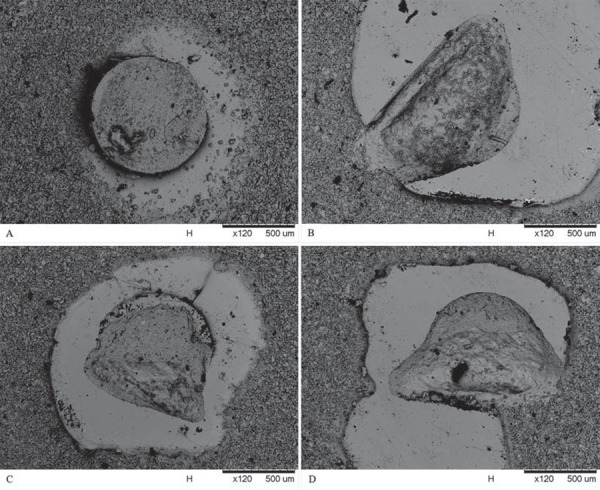
Examples of failure modes of the VL-BC group: A- cohesive failure at resin cement; B- cohesive failure at ceramic substrate; C- mixed failure with predominance of resin cement; D- mixed failure with predominance of ceramic substrate

The failure mode analysis shown in Figures 4 and 5 showed prevalence of cohesive failures in the ceramic substrate (Type III) for the resin cements used in the dual activation mode as well as for the Variolink Veneer light-cured cement. A higher percentage of cohesive failures in resin cement (Type II) or mixed failures with predominance of resin cement (Type IV) were observed for RelyX ARC when using the light-activation mode. No dominant failure mode was verified for the Variolink II in the light-activation mode.

## Discussion

The hypothesis tested was accepted. The dual-cured resin cements showed a higher degree of conversion (DC) and at least similar bond strength values when used in the dual activation mode (base and catalyst) when compared with the resin cements used in the light-activation mode (base paste only). The base paste of both the dual-cured resin cements presented higher DC and bond strength values than the light-cured resin cement tested. Moreover, it is important to note that the base paste of Variolink II showed higher bond strength values than the base paste of RelyX ARC; and to consider that not all resin cements are equal in formulation and may present different curing properties^10^.

When comparing the resin cements using only the base paste in the light-activation mode, there were no significant differences between the DC of RelyX ARC and Variolink II. However, the DC of both resin cements was reduced in the light-activation compared with the dual activation mode. Therefore, the importance of a chemical activation in addition to light curing in dual-cured resin cements must be emphasized. Previous studies have shown the importance of dual-curing resin cements for clinical situations in which the light from the curing unit is not capable of fully reaching all regions of the cavity or preparation, such as in the apical region of root canals during adhesive post luting or the deep internal areas of preparations for indirect adhesive restorations^2,5,6,14^. Thus, when the manufacturer informs that a cement is dual-cured, it is imperative to respect the instruction to perform the double polymerization process, in order to achieve higher DC values and improved properties in daily practice.

Composite-based restorative materials are constantly submitted to agents present in saliva, food and drinks, which may help to degrade the organic matrix^21^. The inadequate polymerization of resin cements, represented by a low DC, may result in increased dissolution of some components in the oral cavity, which may cause pulp sensitivity, in addition to a faster marginal degradation process and early loss of the indirect restoration^13,22^. A high DC ensures the best chemical and physical properties of resin cements^26^.

Variables such as color, thickness and type of ceramic for indirect restorations may interfere in the amount of light reaching the resin-based luting material, influencing the DC^15,25^. Thus, for the DC methodology of this study, a feldspathic ceramic disc was interposed between the resin cement and the light source during curing of the specimens, seeking to emulate what happens in the oral cavity during clinical practice. In this study, it was shown that for cases in which a light-curing resin cement is required, the base pastes of the dual-cured resin cements evaluated used in the light-activation mode showed suitable properties that may allow their use for luting thin ceramic restorations.

Variolink II activated in both dual and light-activation modes showed the highest bond strength values, followed by RelyX ARC in the dual activation mode. In turn, when used in the light-activation mode with the base paste only, RelyX ARC presented values close to the lowest bond strength values, but these were statistically similar to those verified for the light-cured resin cement Variolink Veneer. Despite presenting a suitable bond strength to dental substrates, it is also important for resin cements to offer adequate bond strength to indirect restorations. Bonding to ceramic restorations follows similar principles as those used for bonding to dental tissues. The adhesive or resin cement infiltrates into the roughness created on the surface of these materials, thus enabling micro-mechanical retention^19^.

For this purpose, acid-sensitive ceramics are submitted to surface etching with hydrofluoric acid to increase the surface roughness, improve wettability, surface free energy, and to expose a silicon oxide network^3,7^. Additionally, to assure interaction between the ceramic and resin cement, the application of a coupling agent based on methacryloxypropyltrimethoxysilane allows the formation of covalent bonds between the silica from glass ceramics and the organic matrix from resin cements^8^. The silane coupling agent is also capable to increase wettability and surface energy on the ceramic surface^12,23^.

Regarding the failure mode, dual-activated resin cements (VL-BC and RX-BC) and light-cured resin cement (VL-V) predominantly presented cohesive failures in the ceramic surface of the specimens, characterized as a body failure of the restorative material. This probably occurred because a conventional feldspathic ceramic without any reinforcement was used as the substrate material for bonding tests and also due to the higher bond strengths achieved in these specimens. RX-B specimens presented more cohesive failures involving the resin cement, with body failures in the luting agent. VL-B exhibited heterogeneous failure modes that were not conclusive. No classical adhesive failures were observed for the experimental groups. Therefore, despite the mechanical properties evaluated in this study showing that there were no significant differences between light-cured resin cement and dual-cured resin cements used in the light-activation mode, no favorable failure mode was observed for RX-B and VL-B. Thus, more studies are necessary to evaluate other properties that justify their use in clinical practice.

Although further studies are still needed, the use of dual-cured resin cements used only in the light-activation mode may be an alternative for luting translucent ceramic restorations up to or less than 1.0 mm thick. The degree of conversion and bond strength showed similar behavior between base paste of dual-cured materials and light-cured resin cement. Furthermore, it is important to understand the chemical and mechanical properties of resin cements and the strategies capable of improving these properties, to ensure the success of restorative procedure.
